# Relationship Between Anterior–Posterior Plantar Reaction Force Distribution and Gait Speed in Post-Stroke Patients

**DOI:** 10.3390/jcm15208014

**Published:** 2026-10-16

**Authors:** Daiki Naito, Yusuke Sekiguchi, Keita Honda, Midori Miyagi, Takeshi Yamaguchi, Toshiaki Nishi, Yoshihiro Sasaki, Jun-Ichi Hayasaka, Hide Matsumoto, Yuzuki Nakai, Daiki Haruyama, Satoru Ebihara

**Affiliations:** 1 Department of Rehabilitation Medicine, Graduate School of Medicine, Tohoku University, 1–1 Seiryo-machi, Aoba-ku, Sendai 980-8574, Miyagi, Japan; keita.honda.t2@tohoku.ac.jp (K.H.); midori.miyagi.b5@tohoku.ac.jp (M.M.); satoru.ebihara.c4@tohoku.ac.jp (S.E.); 2 Department of Finemechanics, Graduate School of Engineering, Tohoku University, 6-6-01 Aramaki, Aoba-ku, Sendai 980-8579, Miyagi, Japan; takeshi.yamaguchi.c8@tohoku.ac.jp (T.Y.); toshiaki.nishi.b3@tohoku.ac.jp (T.N.); hide.matsumoto.r4@dc.tohoku.ac.jp (H.M.); nakai.yuzuki.r8@dc.tohoku.ac.jp (Y.N.); 3 Department of Physical Therapy, School of Health Science, International University of Health and Welfare, 2600-1 Kitakanemaru, Otawara 324-8501, Tochigi, Japan; 4 Research Management Center, Organization for Research Promotion, Tohoku University, 1–1 Seiryo-machi, Aoba-ku, Sendai 980-8574, Miyagi, Japan; yusuke.sekiguchi.b2@tohoku.ac.jp; 5 Graduate School of Dentistry, Tohoku University, 4-1 Seiryo-machi, Aoba-ku, Sendai 980-8575, Miyagi, Japan; 6 Department of Rehabilitation, Kumamoto Health Science University, 325 Izumi-machi, Kita-ku, Kumamoto 861-5533, Kumamoto, Japan; 7 Graduate School of Biomedical Engineering, Tohoku University, 6-6-12 Aramaki, Aoba-ku, Sendai 980-8579, Miyagi, Japan; 8 Research Institute for Electromagnetic Materials, 9-5-1 Narita, Tomiya 981-3341, Miyagi, Japan; sasaki@denjiken.ne.jp (Y.S.); hayasakaj@denjiken.ne.jp (J.-I.H.); 9 Department of Physical Therapy, Faculty of Health Sciences, Iryo Sosei University, 5-5-1 Chuodai Iino, Iwaki 970-8551, Fukushima, Japan; haruyama.daiki@isu.ac.jp; 10 Department of Frailty Prevention and Metabolic Regulation, Healthspan Research Center (HeSReC), Tohoku University, 1–1 Seiryo-machi, Aoba-ku, Sendai 980-8574, Miyagi, Japan

**Keywords:** stroke, reaction force distribution, gait speed

## Abstract

**Background:** Gait speed is an important indicator of walking ability in post-stroke patients. Although gait speed is associated with anterior–posterior reaction force (AP RF), how AP RF is distributed across the plantar surface and whether this distribution is associated with gait speed remain unclear. **Methods**: Fifteen post-stroke patients and 18 healthy controls were included. AP RF was measured during walking using a sensor shoe system equipped with four triaxial force sensors located at the heel, first metatarsal (MT1), fifth metatarsal (MT5), and toe regions. AP RF distribution was quantified as the relative contribution of each plantar region and compared among the paretic, non-paretic, and control sides across the stance phase using linear mixed-effects models. Associations between AP RF distribution and gait speed were examined using Spearman’s rank correlation coefficients, with *p*-values adjusted for multiple testing using the Benjamini–Hochberg false discovery rate procedure. **Results**: Significant group × stance phase interactions were observed for the relative contributions of MT1 and MT5. After correction for multiple comparisons, the MT1 contribution at 81–90% of the stance phase was lower on both the paretic and non-paretic sides than on the control side, whereas the MT5 contribution at 1–10% was higher on both sides than on the control side. Total AP RF was also lower on the paretic and/or non-paretic sides than on the control side during several stance phases. On the non-paretic side, the MT5 contribution at 1–10% was negatively correlated with gait speed (rs = −0.66, BH-FDR adjusted *p* = 0.04). **Conclusions**: Post-stroke patients showed altered AP RF distribution during the early and late stance phases compared with healthy controls. Greater MT5 contribution on the non-paretic side during early stance was associated with slower gait speed, suggesting that AP RF distribution may provide additional information for characterizing gait impairment after stroke beyond total AP RF.

## 1. Introduction

Stroke can cause motor impairments that may restrict post-stroke patients from returning to work and participating in social activities [1,2]. Previous studies have shown that approximately half of post-stroke patients do not return to work [1]. It has been reported that successful return to work enhances the quality of life among post-stroke patients, and vocational and social activity return represents a critical goal to be achieved in rehabilitation [3]. Among the numerous factors limiting social and vocational return, stroke-induced gait impairment is a key determinant [4,5]. Gait speed is widely used across numerous studies as a primary indicator of gait ability [6,7,8,9]. In addition, gait speed reflects the life space mobility of post-stroke patients [10]. Therefore, improving gait speed plays a critical role in facilitating social and vocational return.

Previous studies have demonstrated that post-stroke patients exhibit a reduced gait speed compared to healthy subjects [8,11]. Reduced gait speed has been associated with impaired propulsive force during walking [12,13]. Given that the propulsive force generated by post-stroke patients is greater in the non-paretic lower limb compared with the paretic lower limb, the non-paretic limb may play an important compensatory role in propulsion [14]. Furthermore, it has been reported that the propulsive force on the non-paretic side is associated with an increase in gait speed in post-stroke patients [15]. Conversely, it has been demonstrated that the contribution of the non-paretic ankle moment to propulsive force during walking is reduced in post-stroke patients compared to healthy controls [13]. In addition, the plantar pressure trajectory length on the non-paretic lower limb during walking is reduced in post-stroke patients compared with healthy subjects [16]. These findings suggest that although the non-paretic side compensates for propulsion, the control mechanism of this propulsive force may be altered. However, it remains unclear how these forces are distributed within the paretic and non-paretic plantar surfaces based on ankle moments and pressure data.

Addressing this limitation requires measuring the reaction force acting on the plantar surface (RF) during gait and analyzing how walking propulsion is generated through the distribution of this force. However, commonly used force sensors calculate only the total ground reaction force, making it impossible to determine how the RF is distributed within the plantar surface. To overcome this, this study used a sensor shoe system integrated with four tri-axial force sensors in the outsole, which allowed us to determine the distribution of RF [17]. In a previous study involving healthy subjects, this sensor shoe system was used to identify differences in RF distribution during walking between younger and older adults [18]. These findings suggest that RF distribution may reflect differences in gait characteristics. Given that ankle kinematics on both the paretic and non-paretic sides during walking differ between post-stroke patients and healthy controls [19], AP RF distribution may also differ between these populations. Furthermore, examining AP RF distribution may provide additional insight into kinetic factors associated with gait speed that cannot be captured by total RF alone. Therefore, clarifying the relationship between AP RF distribution during walking and gait speed may contribute to a more detailed understanding of the kinetic characteristics associated with gait impairment in post-stroke patients and may provide a basis for future studies investigating rehabilitation strategies to improve gait speed. Thus, the objective of this study was to investigate the relationship between the AP RF distribution during walking and gait speed in post-stroke patients and to identify kinetic factors associated with decreased gait speed. We hypothesized that the heel contribution to AP RF distribution on the non-paretic side during walking would be lower in post-stroke patients compared to healthy subjects.

## 2. Materials and Methods

### 2.1. Participants

This study represents a secondary analysis of a data set previously collected for a separate investigation [20]. A total of 20 post-stroke patients (13 males, age range of 55–88 years) and 20 healthy controls (10 males, age range of 60–77) were included in this study. The inclusion criteria for the post-stroke patients were as follows: (1) hemorrhage or infarction in the supratentorial region on unilateral side by computed tomography (CT) or magnetic resonance imaging (MRI) and diagnosis of cerebral hemorrhage or infarction, (2) patients aged 20 years old or older, and (3) patients who were able to walk in a straight line for 30 consecutive gait cycles at their self-selected comfortable speed without assistance from another person or the use of lower-limb orthoses. The inclusion criteria for controls also included (2) and (3). The exclusion criteria for post-stroke group were as follows: (1) comorbid musculoskeletal disorders that impede walking performance; (2) difficulty in communicating and understanding instructions because of higher brain dysfunction, cognitive decline, or aphasia, which makes executing the study challenging; (3) history of neurological disease; (4) inadequate foot clearance during gait resulting in foot dragging that compromised measurement accuracy. The exclusion criteria for controls were as follows: (1)–(4), and (5) history of falls within the past year. The decision to exclude participants based on inadequate foot clearance was made by the first author during the data-processing stage, before RF calculation and statistical analyses were performed. Thus, the exclusion decision was made without knowledge of the RF outcomes. After recruitment of the post-stroke patients was completed, control participants of similar age were recruited from community-dwelling older adults. Participants provided written informed consent.

### 2.2. Clinical Assessment

For post-stroke patients, motor function was assessed using Fugl–Meyer Assessment of lower extremity (FMA-L) [21] and Stroke Impairment Assessment Set (SIAS) to determine scores for lower extremity motor function (hip flexion test, knee extension test, and foot tap test) [22]. Balance ability was assessed using Berg Balance Scale (BBS) [23].

### 2.3. Gait Assessment

**Figure 1:**
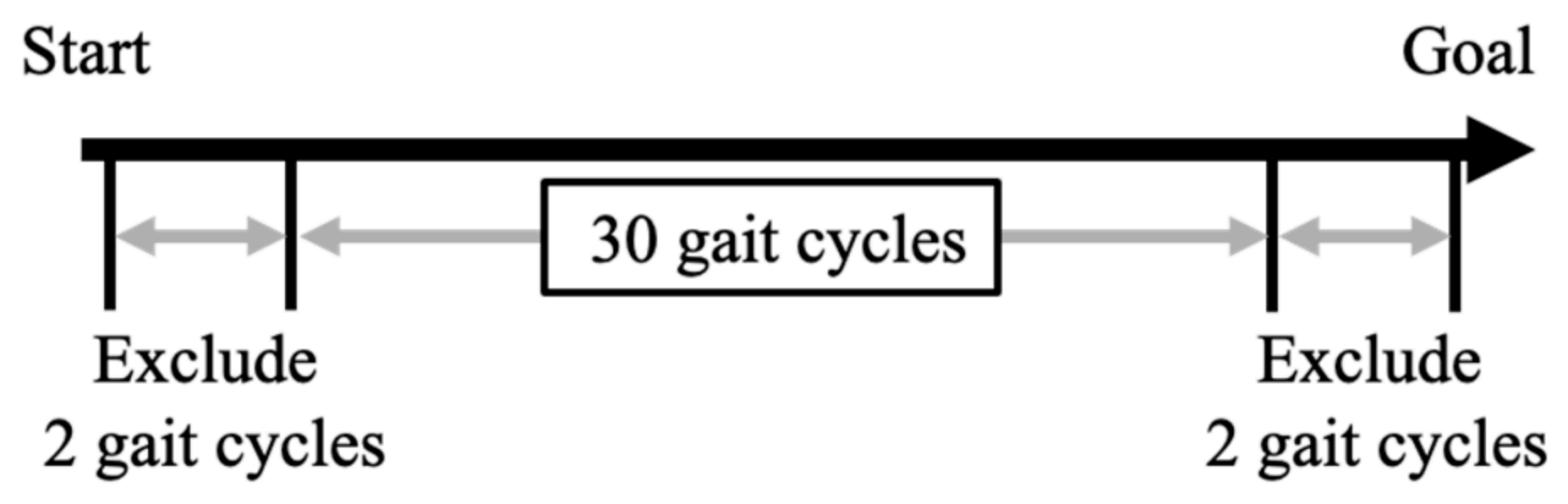
Gait task. Reproduced with permission [20].

The motor task required participants to walk in a straight line for 34 gait cycles at their self-selected comfortable speed. Participants completed one trial of the walking measurement. Thirty consecutive gait cycles were subsequently used for analysis, excluding the initial and final two gait cycles immediately after the start and before the end of walking, respectively [24] (Figure 1). RF values from 30 consecutive gait cycles were averaged within each participant, and the participant-level mean values were used for the statistical analyses. Thus, the experimental unit was the participant.

### 2.4. Shoe Sensor System

**Figure 2:**
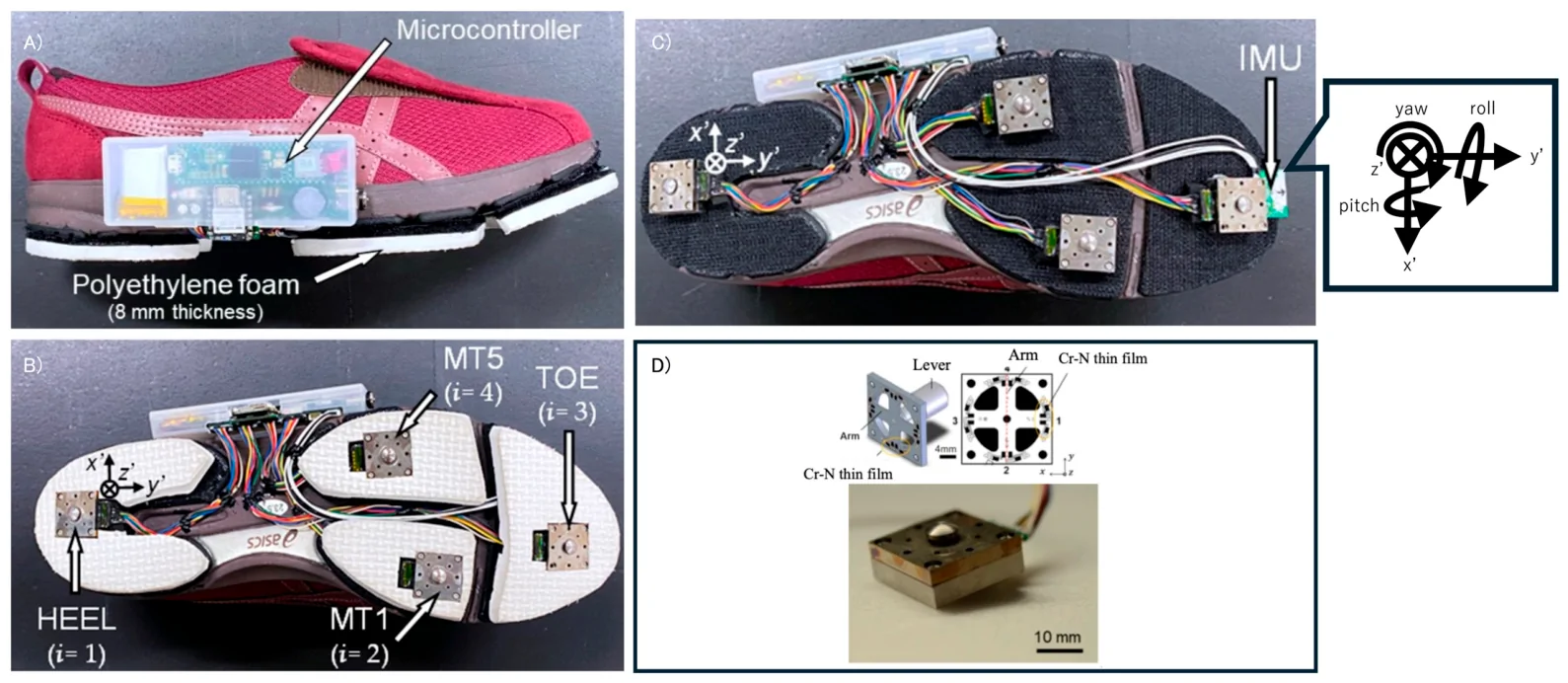
The shoe sensor used for gait measurement. (**A**) The external appearance of the sensor shoe. (**B**) Locations of the reaction force sensors embedded in the sole. HEE, heel; MT1, first metatarsal region; MT5, fifth metatarsal region; TOE, toes. (**C**) An illustration of the inertial sensors integrated into the sole. IMU: Inertial measurement unit. (**D**) Internal structure of a single sensor unit. Reproduced with permission [20].

This study was a secondary analysis of data previously collected and analyzed in our previous study [20]. In contrast to the previous study, the present study focused on RF distribution as the primary outcome. The analyses and findings regarding RF distribution and its association with gait speed presented in the current study were not reported in our previous study. RF during straight walking was assessed using a sensor shoe system [17,18] (Figure 2). The sensor shoe system is equipped with four triaxial force sensors located at the heel, first and fifth metatarsal heads, and toe, allowing RF to be measured outside the laboratory setting [17,18]. Conventionally, ground reaction forces are measured using force plates that determine applied loads from signals generated by strain gauges. In contrast, the sensor shoe system directly measures the forces applied to the plantar surface via sensors integrated into the shoe sole. Accordingly, we defined these forces as “RF acting on the plantar surface”. Because the force measured by the sensor shoe system depends on discrete sensor locations on the sole, the total RF measured by each sensor tends to be lower than the ground reaction force measured across a force plate. Nevertheless, high intra-rater reliability has been confirmed for the RF values obtained during walking in post-stroke patients [20]. Each force sensor consists of a stainless-steel housing and a force-sensing lever (Research Institute for Electromagnetic Materials, Tomiya, Miyagi, Japan). RF was measured along the anterior–posterior (AP), vertical (V), and medial–lateral axes. RF data from both the paretic and non-paretic sides of post-stroke patients were used in the analysis. For healthy controls, RF data from the non-dominant limb (the left limb in all participants) were analyzed. Data from the non-dominant limb were used because this limb has been reported to exhibit smaller changes in GRF in response to variations in walking speed [25]. To minimize the potential influence of walking speed, data from the non-dominant limb were therefore used in the present study. Furthermore, to minimize the influence of differences in footwear on gait mechanics, healthy controls wore bilateral sensor shoes during the measurements. The sampling frequency was 400 Hz. The maximum measurement range of the force sensor was ±500 N for the AP and medial–lateral axes and ±1000 N for the V axis. The ICC for RF during the 1–10% stance phase was 0.49, indicating low reliability. This is likely due to the influence of foot contact points during heel strike. Therefore, the RF data for this segment were interpreted with caution.

An inertial measurement unit (IMU, 9-DOF Absolute Orientation IMU Fusion Breakout-BNO055; Adafruit, NY, USA), with a sampling frequency of 70 Hz, was mounted at the toe of the shoe. The IMU was housed in polyethylene foam embedded in the shoe sole to protect it from external impacts. Both the force sensors and IMU were connected to a microcontroller (Teensy 3.6, SparkFun Electronics^®^, Niwot, CO, USA). The microcontroller was connected to a trigger that was activated by pressing a switch. Measurement data were recorded on a secure digital card.

### 2.5. Data Processing

Data analysis in this study was performed using MATLAB version R2023b (the MathWorks, Inc., Natick, MA, USA, https://www.mathworks.com (accessed on 12 February 2024)). Data processing followed the methods described by Yamaguchi et al. and Matsumoto et al., who used the same sensor shoe system [17,18]. RF data obtained from 30 consecutive gait cycles were processed for each participant and subsequently averaged within each participant for statistical analysis. Prior to participant testing, the sensor shoes were connected to a personal computer and placed on a level surface for calibration. Acceleration data were calibrated using a Newton iterative optimization algorithm [18]. This algorithm determined the calibration coefficients and bias values by minimizing the error between the resultant vector of the triaxial acceleration measured by the IMU under static conditions and gravitational acceleration (9.8 m/s^2^). The Newton iterative optimization algorithm is an iterative method that calculates the solution that minimizes the calibration coefficients and bias values. This minimizes the error between the resultant of the three-axis acceleration data and the acceleration due to gravity (9.8 m/s^2^). Using this algorithm, the sensor shoes were calibrated when placed on a level surface without a participant so that the resultant three-axis acceleration equaled the acceleration due to gravity (9.8 m/s^2^). The corrected acceleration values for each axis (*a*_𝓍0_, *a*_𝓎0_, *a*_𝓏0_) were thus determined. Next, the initial angles of the IMU *θ*_0_ around the 𝓍′-axis and *φ*_0_ around the 𝓎′-axis at the initial static position of the IMU sensor were calculated using calibrated acceleration data (*a*_𝓍0_, *a*_𝓎0_, *a*_𝓏0_) according to the following equations:

$$\theta_{0}=-\arctan\left(\frac{a_{z0}}{a_{x0}}\right)$$

$$\varphi_{0}=\arctan(\frac{a_{x0}}{\sqrt{a_{y0^{2}}+a_{z0^{2}}}})$$

The initial angle around the 𝓏′-axis (*ψ*_0_) was set to 0°. The IMU angles *θ*, *φ*, and *ψ* around each axis during walking were obtained by sequentially adding the Euler angles calculated from the quaternion output of the IMU to the initial angles *θ*_0_, *φ*_0_, and *ψ*_0_.

**Figure 3:**
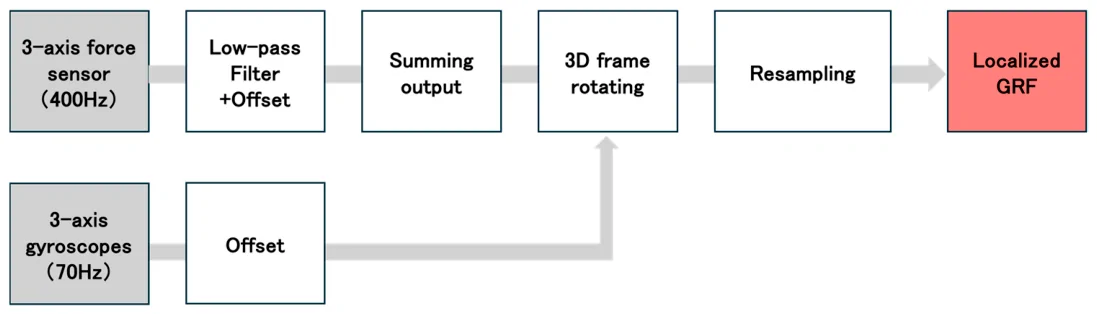
The algorithm for the reaction force acting on the plantar surface (RF) data analysis. A flowchart illustrating the RF data analysis process. The RF data were first corrected using the angular velocity data measured by the inertial sensors, and then, the final values were calculated. Reproduced with permission [20].

Figure 3 shows the analytical algorithm used to calculate the RF obtained from the individual sensors at each location [17,18]. We analyzed the medial–lateral RF (*F*_𝒳′𝒾_), the AP RF (*F*_𝓎′𝒾_), and V RF (*F*_𝓏′𝒾_) recorded during the stance phase. The subscript 𝒾 denotes the location of each sensor on the foot sole: 1 = heel, 2 = first metatarsal head, 3 = fifth metatarsal head, and 4 = toe. To remove noise from the data, a fourth-order Butterworth low-pass filter with a cutoff frequency of 50 Hz was applied. Next, the RF data from each sensor location (ℱ_𝓍′𝒾_, ℱ_𝓎′𝒾_, ℱ_𝓏′𝒾_) were transformed into the global horizontal and vertical coordinate system (ℱ_𝒳𝒾_, ℱ_𝒴𝒾_, ℱ*_z_*_𝒾_) using the following equations [18]:

$$\left[\begin{array}{c} F_{\mathrm{xi}}\\ F_{\mathrm{yi}}\\ F_{\mathrm{zi}} \end{array}\right]=\left[\begin{array}{c} \begin{array}{ccc} \cos\varphi & 0 & \sin\varphi \\ 0 & 1 & 0\\ -\sin\varphi & 0 & \cos\varphi \end{array} \end{array}\right]\left[\begin{array}{ccc} 1 & 0 & 0\\ 0 & \cos\theta & -\;\sin\theta \\ 0 & \sin\theta & \cos\theta \end{array}\right]\left[\begin{array}{c} F_{x^{\prime }i}\\ F_{x^{\prime }i}\\ F_{z^{\prime }i} \end{array}\right]$$

The RF for the entire sensor shoe (ℱ𝓍, ℱ𝓎, ℱ𝓏) was calculated by summing the forces from all sensor locations using the following equation [18]:

$$\left[\begin{array}{c} F_{x}\\ F_{y}\\ F_{z} \end{array}\right]=\sum_{i=1}^{i=4}\left[\begin{array}{c} F_{\mathrm{xi}}\\ F_{\mathrm{yi}}\\ F_{\mathrm{zi}} \end{array}\right]$$

**Figure 4:**
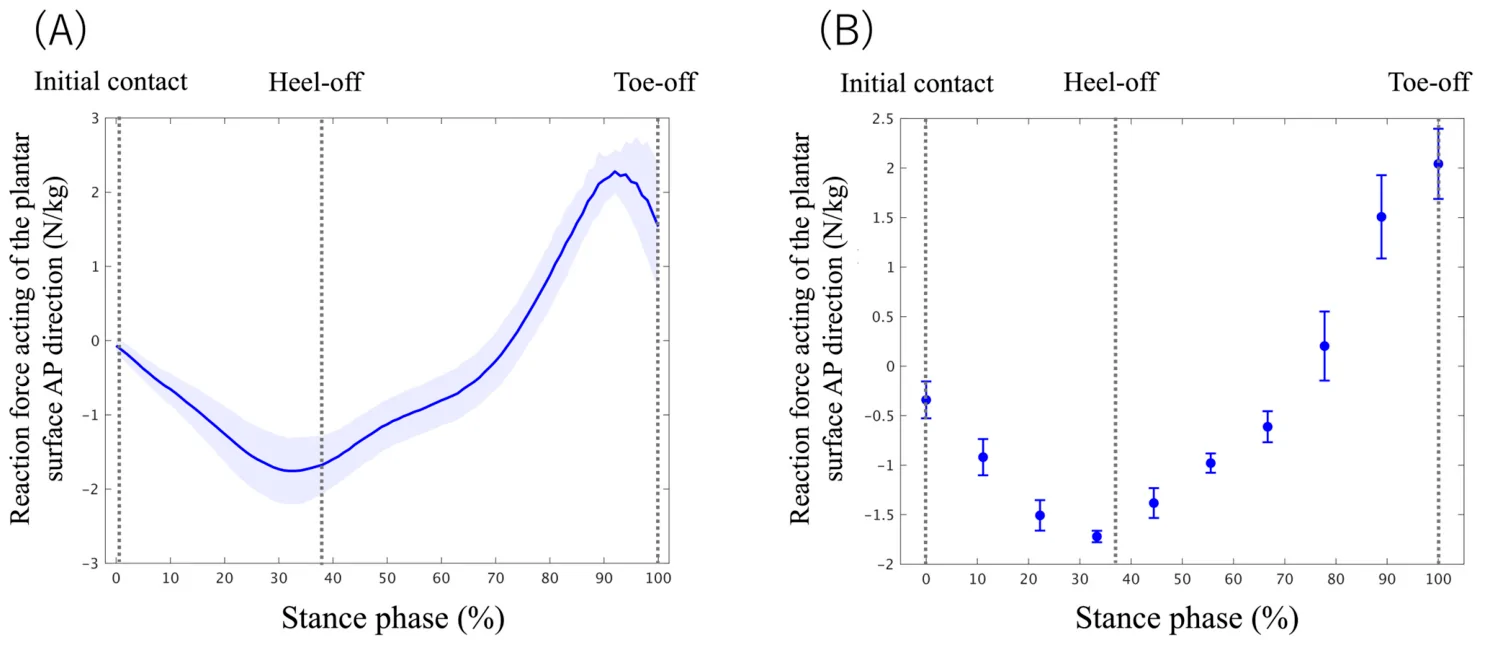
Changes in total anterior–posterior reaction force acting on the plantar surface (RF) during the stance phase, shown as a representative example of a healthy individual. The dashed lines indicate the stance phase events (heel-off, toe-off). (**A**) Time course of RF normalized by body weight (*y*-axis) over the stance phase (*x*-axis). The shaded area indicates the standard deviation of RF. (**B**) RF magnitude averaged for every 10% of the stance phase. The error bars indicate the standard deviation of RF for each phase.

The stance phase of walking was identified using V RF (ℱ*_z_*) from each sensor. The onset of the stance phase was defined as the moment when the V RF (ℱ*_z_*) exceeded 15 N, and the termination of the stance phase was defined as the moment when the V RF (ℱ*_z_*) dropped below 15 N [17]. Previous studies involving stroke survivors have defined the stance phase using force thresholds ranging from 10 N to 50 N [26,27,28]. Therefore, the 15 N threshold used in the present study was expected to accurately detect the stance phase. The RFs calculated for each sensor during the gait cycle were then time-normalized from 0% at heel strike to 100% at toe-off, and the data were subsequently normalized to the participant’s body mass. The time-normalized RF data for the stance phase (0% to 100%) were averaged into 10% intervals to facilitate comparisons across the stance phase. This yielded ten data points representing the RF at each 10% interval of the stance phase (Figure 4A,B).

### 2.6. Reaction Force Distribution Across the Plantar Surface

**Figure 5:**
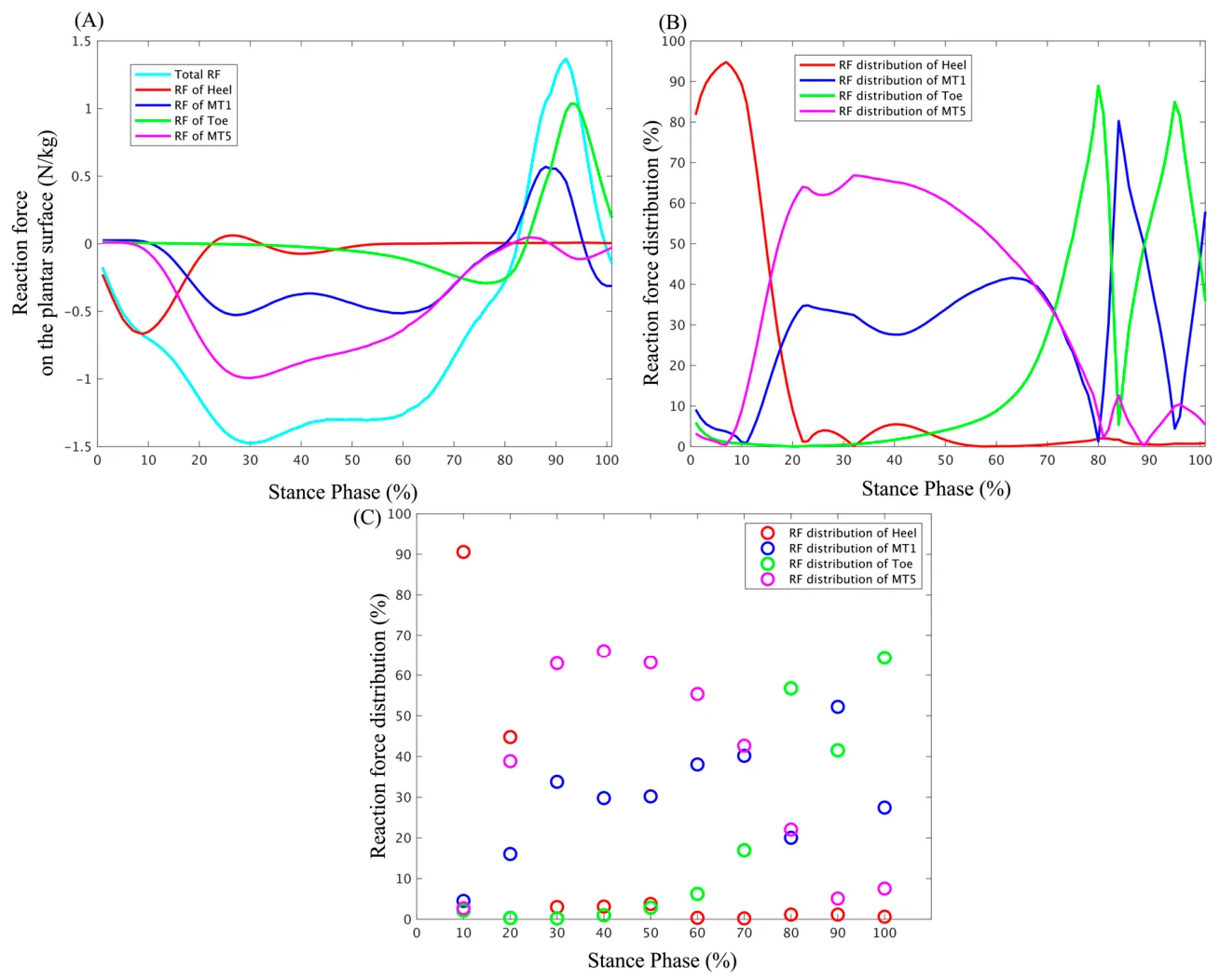
Changes in anterior–posterior (AP) reaction force acting on the plantar surface (RF) distribution during the stance phase, shown as a representative example of a healthy subject. (**A**) Total AP RF and the values from each localized sensor, normalized to body weight, are plotted on the *y*-axis against the time course of the stance phase on the *x*-axis. (**B**) The relative contribution of each plantar location to the sum of the absolute AP RF magnitudes measured across all sensors is plotted on the *y*-axis against the time course of the stance phase on the *x*-axis. (**C**) The relative contribution of each sensor at each 10% interval of the stance phase.

Before calculating the distribution, the measured AP RF was normalized to body mass, with positive and negative values representing propulsive and braking forces, respectively. AP RF distribution was characterized by the relative contribution of each plantar location to the sum of the absolute AP RF magnitudes measured across all sensors. To account for both positive and negative force values, the absolute values of RF were used to calculate the distribution. After calculating the RF distribution, the data were averaged over 10% intervals to facilitate comparisons across the stance phase (Figure 5A–C).

### 2.7. Statistical Analysis

Statistical analyses in this study were performed using JMP Pro 17 (SAS Institute Inc., Cary, NC, USA) and MATLAB version R2023b (The MathWorks, Inc., Natick, MA, USA, https://www.mathworks.com). Before group comparisons, the distribution of the data was verified using the Shapiro–Wilk test, and the homogeneity of variances was checked using Levene’s test. An unpaired *t*-test was used to compare the following between post-stroke patients and controls: age, height, body weight, and gait speed. χ^2^ test was used to compare sex between the groups. Fisher’s exact test was used to compare cane and orthosis usage between the groups.

Linear mixed-effects models (LMMs) were used to examine between-group differences in the total AP RF and the AP RF distribution. Group, stance phase, and the group × stance phase interaction were included as fixed effects, with participant included as a random effect. For variables showing a significant group × stance phase interaction, simple effects of group were subsequently examined separately at each stance phase using LMMs with group as a fixed effect and participant as a random effect. To account for multiple testing across stance phases, the resulting *p*-values were adjusted using the Benjamini–Hochberg false discovery rate (BH-FDR) procedure within each variable. For stance phases showing a significant group effect after BH-FDR correction, post-hoc pairwise comparisons were performed among the paretic, non-paretic, and control sides using model-based contrasts. The *p*-values for the three pairwise comparisons within each significant stance phase were adjusted using the Holm method. Statistical significance was set at *p* < 0.05. For analyses involving multiple comparisons, adjusted *p*-values were used to determine statistical significance. The effect size (partial η^2^) was defined as follows: large (≥0.14), medium (≥0.06) and small (≥0.01) [29]. Sufficient statistical power (1 − β) was defined as ≥0.80 [29].

Spearman’s rank correlation coefficients were used to examine the associations between gait speed and AP RF distribution at stance phases showing significant group differences. A total of four correlation tests were performed, and the *p*-values were adjusted for multiple testing using the Benjamini–Hochberg false discovery rate (BH-FDR) procedure. The correlation coefficients were classified as weak (r < 0.50), moderate (0.50 ≤ r < 0.80), and strong (r ≥ 0.80) [30].

During the preparation of this manuscript, Google Gemini (Google LLC, Mountain View, CA, USA) was used solely to check English grammar and improve linguistic clarity. It was not used for the study design, data collection, data analysis, interpretation of the results, or generation of scientific content. All AI-assisted revisions were reviewed and verified by the authors.

## 3. Results

### 3.1. Participant Characteristics

**Figure 6:**
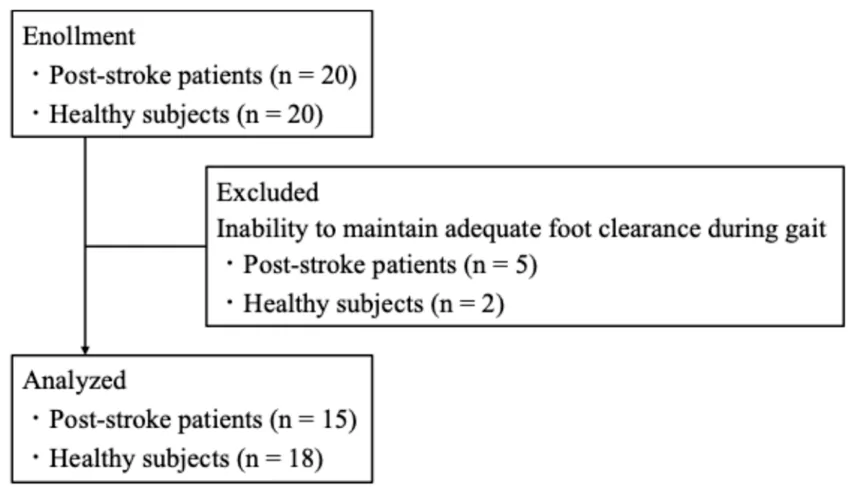
Flowchart of participant selection.

A total of 20 post-stroke patients and 20 healthy controls met the inclusion criteria and participated in the measurements. In the excluded participants, reduced foot clearance was clearly identified by visual observation. Based on the predefined exclusion criterion of inadequate foot clearance, these participants were excluded from the RF calculation and subsequent statistical analyses. Ultimately, 15 post-stroke patients and 18 healthy controls were included in the final analysis (Figure 6). Table 1 summarizes the demographic and clinical characteristics of the post-stroke patients and healthy controls. There were no significant differences between the groups in age, sex ratio, height, or body mass. Cane use was significantly more frequent, and gait speed was significantly lower, in the post-stroke patients than in the healthy controls. Specifically, five post-stroke patients and no healthy controls used a cane during the measurements, whereas one post-stroke patient and no healthy controls regularly used a lower-limb orthosis in daily life. Clinical diagnoses, along with FMA-L, BBS, and SIAS scores for the post-stroke patients, are also presented.

### 3.2. Comparison of AP RF Distribution Across the Stance Phase Among the Paretic Side, Non-Paretic Side and Control Side

Table 2 shows the RF distribution in the three groups. The LMMs revealed significant group × stance phase interactions for MT1 (*F* = 1.66, *p* = 0.04, partial η^2^ = 0.05) and MT5 (*F* = 2.38, *p* < 0.01, partial η^2^ = 0.07), whereas no significant interactions were observed for the Heel or Toe regions. Significant main effects of stance phase were observed for the AP RF distribution of all plantar regions (all *p* < 0.01). Significant main effects of group were observed for the Heel (*F* = 9.43, *p* < 0.01, partial η^2^ = 0.17) and Toe (*F* = 6.03, *p* < 0.01, partial η^2^ = 0.13) (Table 3). Given the significant group × stance phase interactions, phase-specific group differences were subsequently examined for MT1 and MT5. After BH-FDR correction, a significant group difference in the MT1 contribution was observed at 81–90% of the stance phase (adjusted *p* = 0.03), whereas a significant group difference in the MT5 contribution was observed at 0–10% of the stance phase (adjusted *p* = 0.04) (Table 4). Post-hoc pairwise comparisons with Holm correction showed that the MT1 contribution at 81–90% differed significantly between the non-paretic and control sides (adjusted *p* < 0.01) and between the paretic and control sides (adjusted *p* = 0.01). The MT5 contribution at 0–10% differed significantly between the non-paretic and control sides (adjusted *p* < 0.01) and between the paretic and control sides (adjusted *p* = 0.04) (Table 5).

### 3.3. Comparison of Total AP RF Across the Stance Phase Among the Paretic Side, Non-Paretic Side and Healthy Control Side

Table 2 shows the total AP RF in the three groups. For total AP RF, the LMM revealed a significant group × stance phase interaction (*F* = 2.48, *p* < 0.01, partial η^2^ = 0.07), as well as significant main effects of group (*F* = 6.87, *p* < 0.01, partial η^2^ = 0.16) and stance phase (*F* = 48.80, *p* < 0.01, partial η^2^ = 0.44) (Table 3). Phase-specific analyses with BH-FDR correction showed significant group differences at 11–20%, 31–40%, and 41–100% of the stance phase (all adjusted *p* < 0.05) (Table 4). Post-hoc pairwise comparisons with Holm correction showed significantly lower total AP RF on the non-paretic side than on the control side at 11–20%, 31–40%, and 41–100% of the stance phase (all adjusted *p* < 0.05). Total AP RF was also significantly lower on the paretic side than on the control side at 41–100% of the stance phase (all adjusted *p* < 0.05) (Table 5).

### 3.4. Correlations Between AP RF Distribution and Gait Speed

**Figure 7:**
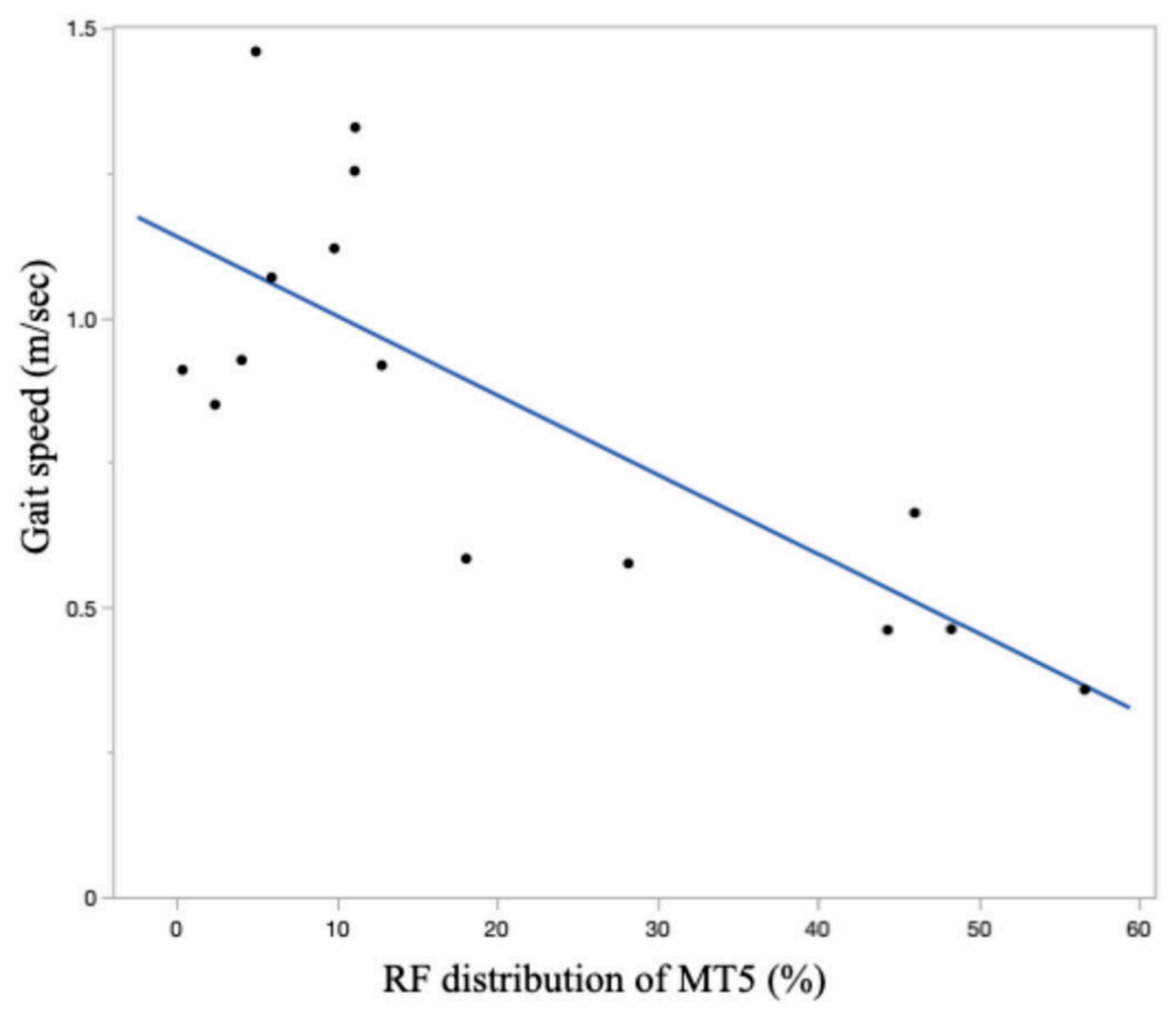
Relationship between MT5 contribution on the non-paretic side and gait speed during 0–10% stance phase.

Spearman’s rank correlation analysis revealed a significant negative correlation between gait speed and the MT5 contribution at 0–10% of the stance phase on the non-paretic side (r_s_ = −0.66, BH-FDR-adjusted *p* = 0.04). No significant correlations were observed for MT5 on the paretic side or for MT1 on either the paretic or non-paretic side after BH-FDR correction for multiple testing (Table 6, Figure 7).

## 4. Discussion

The present study investigated RF distribution during gait in post-stroke patients. The main findings were that RF distribution differed among the paretic side, non-paretic side, and controls during the early and late stance phases. Total AP RF also differed among the groups across the stance phase. Furthermore, MT5 contribution during early stance on the non-paretic side was associated with gait speed. These findings suggest that RF distribution may provide additional insight into gait characteristics after stroke beyond that provided by total AP RF alone. In particular, identifying specific features of RF distribution associated with gait speed may contribute to a more detailed understanding of the kinetic characteristics associated with gait impairment in post-stroke patients. Importantly, these findings regarding RF distribution and its association with gait speed were not reported in our previous study and represent a novel aspect of the present secondary analysis.

### 4.1. Characteristics of AP RF Distribution During Gait in Post-Stroke Patients

The present study identified characteristic alterations in RF distribution in post-stroke patients during 0–10% and 81–90% of the stance phase, corresponding approximately to the double-support periods. During late stance, the contribution of MT1 was lower on both the paretic and non-paretic sides than on the control side. Total AP RF was also lower on both sides, indicating reduced forward propulsion in post-stroke patients. Previous studies have suggested that plantar pressure around the first metatarsal head contributes to forward propulsion during gait [31]. The concurrent reductions in MT1 contribution and total AP RF observed in the present study are consistent with these findings and suggest impaired force generation through the medial forefoot during late stance. In contrast, during early stance, the contribution of MT5 was greater on the paretic side than on the control side, indicating preferential loading of the anterolateral plantar region. This finding is consistent with previous reports of increased anterolateral foot loading on the paretic side after stroke [32]. Notably, a similar increase in MT5 contribution was also observed on the non-paretic side. In normal gait, sufficient propulsion during late stance facilitates progression of the limb into the swing phase [33]. The reduced MT1 contribution on the paretic side may therefore reflect insufficient propulsion and an impaired transition into the swing phase. To facilitate paretic limb advancement, compensatory strategies such as lateral displacement of the center of pressure on the non-paretic side, together with hip hiking and circumduction of the paretic limb, may be required [34,35]. Accordingly, the increased MT5 contribution on the non-paretic side may reflect a compensatory lateral loading strategy that facilitates paretic limb advancement. Collectively, these findings suggest that RF distribution during gait in post-stroke patients is particularly altered during the double-support periods. These alterations in RF distribution complement previous evidence suggesting that reduced RF variability during late stance may represent a strategy to enhance gait stability after stroke [20]. These findings highlight the importance of further investigating double-support mechanics to better characterize post-stroke gait dysfunction.

### 4.2. Relationship Between RF Distribution on the Non-Paretic Side and Gait Speed

The results of this study suggest that early anterolateral plantar loading on the non-paretic side during walking may be associated with reduced gait speed in post-stroke patients, contrary to our hypothesis. Previous studies have demonstrated that braking force plays an important role in gait speed control after stroke [36,37,38] and that increasing gait speed requires both braking force during early stance and propulsive force during terminal stance [39,40,41,42]. Given that gait speed after stroke is associated with propulsive force generated by the non-paretic side [14], the non-paretic limb may also play an important role in generating braking force for gait speed control. Although total AP RF did not differ significantly among the groups during 0–10% of the stance phase, AP RF distribution differed significantly. Specifically, the non-paretic side showed altered RF distribution in the anterolateral plantar region during early stance, which may reflect earlier loading of this region following initial contact. Previous research has shown that kinematic variations at initial contact are closely associated with gait speed after stroke [43]. In particular, a forefoot contact pattern has been associated with reduced gait speed and altered vertical ground reaction forces [43]. Taken together, these findings suggest that gait speed may be associated not only with the magnitude of braking force but also with where this force is applied across the plantar surface. Thus, the contact and loading pattern of the non-paretic foot may represent a potential target for future interventional studies investigating gait speed after stroke. However, the reliability of measurements during 0–10% of the stance phase has been reported to be relatively low. Therefore, these findings should be interpreted with caution.

### 4.3. Limitations of the Study and Future Research

Several limitations of this study should be noted. First, the post-stroke patients in this study had relatively mild motor paralysis, balance impairment and gait impairment; therefore, caution is warranted when applying these findings to individuals with more severe impairments. Second, cane use was not controlled during gait measurement and was limited to post-stroke patients. Therefore, the gait conditions were not fully comparable between the post-stroke patients and controls, warranting cautious interpretation of the between-group differences. In particular, cane use has been reported to improve gait speed in post-stroke patients who walk at 0.4–0.8 m/s [44]. Future studies should standardize cane-use conditions and include healthy controls under comparable gait conditions. Third, because the sensor shoe system used a fixed size, the absolute positions of the sensors could not be standardized according to each participant’s foot size. Nevertheless, the ability to measure RF during gait in real-world environments outside the laboratory represents an important methodological advantage of the sensor shoe system. Fourth, for healthy controls, data from only the left side (non-dominant side) were used. However, it has been reported that ground reaction force during gait is symmetrical in healthy older adults with a relatively fast gait speed (1.25 m/s) [45]. Therefore, using data solely from the left side in healthy controls is expected to have minimal impact on the between-group comparison. Fifth, approximately 25% of the post-stroke patients enrolled in this study were excluded from the final analysis. Therefore, caution is warranted when generalizing the present findings to post-stroke patients with reduced foot clearance. However, the physical function of the excluded patients appeared to be generally similar to that of the patients included in the final analysis, and none of the excluded patients used a cane or orthosis during walking. Nevertheless, the exclusion of these patients indicates that the present study may not fully capture the variability in gait patterns among post-stroke patients, even among those with relatively mild functional impairments. Future studies should further refine the measurement and analytical methods to enable the investigation of a broader range of gait impairments and gait patterns in post-stroke patients. Finally, this study was a secondary analysis of a previously collected dataset, precluding an a priori sample size calculation specifically targeting the relationship between AP RF distribution and gait speed. In addition, participant dropouts from the original cohort resulted in a relatively small final sample. The available sample size provided sufficient power to detect only large effect sizes; therefore, smaller associations may have been missed due to Type II error. Future prospective studies with larger, adequately powered samples are warranted to confirm these findings.

Future studies with larger sample sizes of both patients and controls, incorporating bilateral data from healthy controls, may further clarify RF distribution patterns after stroke.

## 5. Conclusions

This study demonstrated differences in AP RF distribution during gait between post-stroke patients and healthy controls, particularly during the early and late stance phases. Post-stroke patients showed greater MT5 contribution during early stance and lower MT1 contribution during late stance than healthy controls. In addition, greater MT5 contribution on the non-paretic side during early stance was associated with slower gait speed. These findings suggest that AP RF distribution across the plantar surface may provide additional information for characterizing gait impairment after stroke beyond total AP RF. Further studies, including longitudinal studies, are needed to better understand the relationship between AP RF distribution and gait speed.

## Figures and Tables

**Table 1:** Characteristics of post-stroke patients and healthy controls.

	Post-Stroke Patients	Control	Test Statistics	*p* Value
Number	15	18		
Age (years) ^a,c^	70.20 (7.78)	68.72 (5.88)	0.62	0.73
Sex (male/female) ^d^	12/3	10/8	2.27	0.13
Height (cm) ^a,c^	158.93 (11.55)	161.41 (9.06)	0.47	0.50
Weight (kg) ^a,c^	57.13 (9.51)	55.82 (10.66)	0.15	0.70
Cane (T-cane/none) ^e^	5/10	0/18	-	0.03 *
Gait speed (m/s) ^a,c^	0.86 (0.31)	1.24 (0.11)	19.82	<0.01 *
Orthosis (AFO/none) ^e^	1/14	0/18	-	0.45
Diagnosis				
Infarction ^e^	9	-	-	-
Hemorrhage ^e^	6	-	-	-
Paretic side ^e^			-	
Right ^e^	8	-	-	-
Left ^e^	7	-	-	-
Time post-stroke (days) ^b^	86 (110–62)	-	-	-
Fugl Meyer Assessment ^b^	33 (34–28)	-	-	-
Berg Balance scale ^b^	53 (56–50)	-	-	-
Stroke Impairment Assessment Set (0/1/2/3/4/5) ^f^		-	-	-
Hip flexion test	0/0/0/1/3/11	-	-	-
Knee extension test	0/0/0/2/4/9	-	-	-
Ankle dorsiflexion test	0/0/0/0/4/11	-	-	-

^a^: Mean (standard deviation), ^b^: Median (Interquartile Range), ^c^: Unpaired *t*-test, ^d^: Chi-square test, ^e^: Fisher’s exact test, ^f^: Number (percentage), *: *p* < 0.05.

**Table 2:** Total anterior–posterior reaction force and relative contributions of plantar regions during walking among the paretic, non-paretic, and control sides.

Stance Phase	Paretic Side	Non-Paretic Side	Control
Number	15		18
Total AP RF			
0–10%	−0.56 (0.51)	−0.53 (0.33)	−0.29 (0.40)
11–20%	−1.11 (0.71)	−1.19 (0.55)	−0.52 (0.57)
21–30%	−1.27 (1.02)	−1.44 (0.71)	−0.65 (0.60)
31–40%	−1.41 (1.01)	−1.52 (0.80)	−0.61 (0.63)
41–50%	−1.43 (1.13)	−1.49 (0.89)	−0.40 (0.65)
51–60%	−1.49 (1.23)	−1.48 (1.10)	−0.24 (0.79)
61–70%	−1.52 (1.43)	−1.41 (1.33)	0.06 (1.09)
71–80%	−1.07 (1.54)	−1.15 (1.41)	0.67 (1.31)
81–90%	0.08 (1.20)	−0.22 (0.99)	1.26 (1.00)
91–100%	0.40 (0.48)	0.45 (0.78)	1.07 (0.65)
Relative contribution of Heel			
0–10%	56.67 (32.69)	51.42 (27.32)	57.82 (27.41)
11–20%	40.60 (32.93)	38.36 (22.26)	36.85 (19.70)
21–30%	24.41 (26.30)	22.60 (21.45)	10.45 (9.43)
31–40%	18.49 (20.59)	19.37 (23.59)	6.32 (5.94)
41–50%	15.09 (17.62)	16.42 (24.07)	5.38 (6.58)
51–60%	10.01 (14.63)	12.83 (23.43)	2.02 (3.03)
61–70%	5.95 (11.25)	10.70 (20.10)	1.06 (1.05)
71–80%	3.87 (8.09)	7.82 (14.30)	1.35 (2.73)
81–90%	3.44 (5.01)	8.03 (18.84)	0.83 (1.58)
91–100%	4.23 (7.29)	11.83 (27.17)	0.78 (1.26)
Relative contribution of MT1			
0–10%	15.22 (17.63)	16.53 (17.79)	14.46 (15.32)
11–20%	17.95 (13.21)	15.56 (12.32)	15.75 (17.02)
21–30%	21.82 (13.84)	22.78 (12.86)	24.51 (12.64)
31–40%	25.72 (14.49)	26.85 (15.03)	26.45 (9.17)
41–50%	28.41 (13.59)	29.08 (16.97)	28.93 (9.55)
51–60%	32.81 (12.52)	32.61 (17.70)	30.78 (9.78)
61–70%	34.67 (11.20)	36.01 (17.28)	35.08 (9.95)
71–80%	34.52 (15.60)	38.27 (16.69)	39.03 (11.77)
81–90%	37.15 (17.94)	32.87 (20.95)	51.38 (9.86)
91–100%	21.82 (9.30)	26.73 (23.22)	33.38 (11.99)
Relative contribution of Toe			
0–10%	16.71 (21.72)	8.51 (9.75)	22.03 (24.62)
11–20%	10.03 (16.69)	3.25 (4.53)	15.89 (20.71)
21–30%	12.57 (18.95)	3.51 (5.97)	13.52 (18.79)
31–40%	10.74 (12.55)	3.56 (5.29)	12.61 (14.25)
41–50%	8.87 (9.02)	4.53 (3.43)	11.70 (16.18)
51–60%	10.91 (9.37)	8.62 (8.81)	11.18 (16.11)
61–70%	17.03 (12.61)	13.38 (9.27)	12.50 (9.01)
71–80%	30.17 (19.59)	23.41 (13.35)	25.48 (18.58)
81–90%	45.04 (23.41)	43.28 (23.15)	28.91 (12.06)
91–100%	63.51 (16.01)	55.54 (26.43)	56.43 (13.42)
Relative contribution of MT5			
0–10%	16.92 (14.83)	20.25 (19.22)	5.68 (4.23)
11–20%	33.75 (18.79)	39.62 (19.40)	31.51 (15.71)
21–30%	43.02 (19.58)	50.26 (18.67)	51.52 (16.72)
31–40%	46.72 (18.40)	51.02 (19.87)	54.62 (11.63)
41–50%	49.13 (15.63)	51.19 (20.33)	53.99 (14.57)
51–60%	47.29 (12.48)	47.30 (20.55)	56.03 (13.36)
61–70%	42.87 (13.64)	41.25 (20.96)	51.36 (11.43)
71–80%	31.41 (13.89)	31.50 (17.72)	34.14 (15.20)
81–90%	14.55 (14.85)	16.48 (15.97)	18.88 (11.98)
91–100%	10.78 (7.39)	6.93 (5.07)	9.42 (3.75)

Mean (standard deviation), MT1: The first metatarsal region, MT5: The fifth metatarsal region.

**Table 3:** Comparison of the relative contributions to anterior–posterior reaction force distribution during walking among the paretic, non-paretic, and control sides.

Effect	*F*	*p* Value	Partial η^2^	1 − β
Relative contribution of Heel				
group	9.43	<0.01 *	0.17	0.99
Phase	41.18	<0.01 *	0.40	1.00
group × phase	0.41	0.99	0.01	0.30
Relative contribution of MT1				
group	1.72	0.19	0.04	0.36
Phase	28.86	<0.01 *	0.32	1.00
group × phase	1.66	0.04 *	0.05	0.97
Relative contribution of Toe				
group	6.03	<0.01 *	0.13	0.90
Phase	54.31	<0.01 *	0.47	1.00
group × phase	1.25	0.21	0.04	0.85
Relative contribution of MT5				
group	2.67	0.08	0.06	0.60
Phase	70.85	<0.01 *	0.53	1.00
group × phase	2.38	<0.01 *	0.07	1.00
Total RF				
group	6.87	<0.01 *	0.16	0.94
Phase	48.80	<0.01 *	0.44	1.00
group × phase	2.48	<0.01 *	0.07	0.99

MT1: The first metatarsal region, MT5: The fifth metatarsal region, RF: reaction force acting on the plantar surface, 1 − β: statistical power. Group, phase, and group × phase were included as fixed effects, with participant as a random effect. *: *p* < 0.05.

**Table 4:** Phase-specific group differences in total anterior–posterior reaction force and relative contributions of plantar regions during walking.

	*F*	Raw *p*-Value	BH-FDR *p*-Value	Partial η^2^
Relative contribution of MT1				
0–10%	0.07	0.94	0.95	0.00
11–20%	0.61	0.55	0.95	0.04
21–30%	0.29	0.75	0.95	0.01
31–40%	0.32	0.73	0.95	0.01
41–50%	0.54	0.59	0.95	0.02
51–60%	0.66	0.52	0.95	0.03
61–70%	0.05	0.95	0.95	0.00
71–80%	0.26	0.77	0.95	0.01
81–90%	6.33	0.00	0.03 *	0.17
91–100%	3.84	0.03	0.13	0.11
Relative contribution of MT5				
0–10%	6.09	0.00	0.04 *	0.21
11–20%	1.14	0.33	0.41	0.04
21–30%	1.51	0.23	0.33	0.06
31–40%	2.23	0.12	0.24	0.10
41–50%	1.91	0.16	0.27	0.08
51–60%	3.95	0.03	0.09	0.14
61–70%	3.99	0.02	0.09	0.12
71–80%	0.82	0.45	0.49	0.03
81–90%	0.63	0.53	0.53	0.02
91–100%	2.89	0.06	0.16	0.09
Total RF				
0–10%	1.04	0.36	0.36	0.05
11–20%	3.79	0.03	0.04 *	0.16
21–30%	3.14	0.05	0.06	0.14
31–40%	3.55	0.04	0.04 *	0.18
41–50%	4.38	0.02	0.04 *	0.21
51–60%	4.17	0.02	0.04 *	0.20
61–70%	4.42	0.02	0.04 *	0.20
71–80%	6.16	0.00	0.01 *	0.24
81–90%	7.80	0.00	0.01 *	0.26
91–100%	6.61	0.00	0.01 *	0.18

MT1: The first metatarsal region, MT5: The fifth metatarsal region, RF: Reaction force acting on the plantar surface. 1 − β: statistical power. BH-FDR: Benjamini–Hochberg false discovery rate. *: *p* < 0.05.

**Table 5:** Post-hoc pairwise comparisons of total anterior–posterior reaction force and relative contributions of plantar regions.

Phase	Group 1	Group 2	*F*	Raw *p*	Holm *p*
Relative contribution of MT1					
81–90%	Nonparetic side	Control	10.54	<0.01 *	0.01 *
	Nonparetic side	Paretic side	0.13	0.72	0.72
	Control	Paretic side	8.30	0.01 *	0.01 *
Relative contribution of MT5					
0–10%	Nonparetic side	Control	12.15	<0.01 *	<0.01 *
	Nonparetic side	Paretic side	1.80	0.19	0.19
	Control	Paretic side	5.37	0.02 *	0.04 *
Total RF					
11–20%	Nonparetic side	Control	7.54	0.01 *	0.02 *
31–40%	Nonparetic side	Control	7.00	0.01 *	0.03 *
41–50%	Nonparetic side	Control	8.46	0.01 *	0.02 *
	Control	Paretic side	6.66	0.01 *	0.03 *
51–60%	Nonparetic side	Control	7.89	0.01 *	0.02 *
	Control	Paretic side	6.69	0.01 *	0.03 *
61–70%	Nonparetic side	Control	7.75	0.01 *	0.02 *
	Control	Paretic side	7.51	0.01 *	0.02 *
71–80%	Nonparetic side	Control	11.00	<0.01 *	<0.01 *
	Control	Paretic side	8.82	<0.01 *	0.01 *
81–90%	Nonparetic side	Control	14.77	<0.01 *	<0.01 *
	Control	Paretic side	8.62	<0.01 *	0.01 *
91–100%	Nonparetic side	Control	8.31	0.01 *	0.01 *
	Control	Paretic side	11.30	<0.01 *	<0.01 *

MT1: The first metatarsal region, MT5: The fifth metatarsal region, RF: Reaction force acting on the plantar surface, *: *p* < 0.05.

**Table 6:** Correlations between anterior–posterior reaction force distribution and gait speed.

Stance Phase	r_s_	*p*-Value	BH-FDR *p*-Value
MT1			
Paretic side			
81–90%	−0.43	0.11	0.22
Non-paretic side			
81–90%	0.13	0.66	0.66
MT5			
Paretic side			
0–10%	−0.28	0.31	0.41
Non-paretic side			
0–10%	−0.66	0.01 *	0.04 *

MT1: The first metatarsal region, MT5: the fifth metatarsal region, *: *p* < 0.05.

## Data Availability

The data, including graphs, within this paper are available from the corresponding author upon reasonable request.
